# *Orientia tsutsugamushi* dynamics in vectors and hosts: ecology and risk factors for foci of scrub typhus transmission in northern Thailand

**DOI:** 10.1186/s13071-021-05042-4

**Published:** 2021-10-18

**Authors:** Ivo Elliott, Neeranuch Thangnimitchok, Kittipong Chaisiri, Tri Wangrangsimakul, Piangnet Jaiboon, Nicholas P. J. Day, Daniel H. Paris, Paul N. Newton, Serge Morand

**Affiliations:** 1 Lao-Oxford-Mahosot Hospital-Wellcome Trust Research Unit, Microbiology Laboratory, Mahosot Hospital, Vientiane, Lao PDR; 2 Centre for Tropical Medicine and Global Health, Nuffield Department of Medicine, University of Oxford, Oxford, UK; 3 Faculty of Tropical Medicine, Mahidol University, Bangkok, Thailand; 4 Mahidol-Oxford Tropical Medicine Research Unit, Faculty of Tropical Medicine, Mahidol University, Bangkok, Thailand; 5 Department of Medicine, Swiss Tropical and Public Health Institute, Basel, Switzerland; 6 Department of Clinical Research, University of Basel, Basel, Switzerland; 7 CNRS ISEM–CIRAD ASTRE, Faculty of Veterinary Technology, Kasetsart University, Bangkok, Thailand

**Keywords:** Scrub typhus, *Orientia tsutsugamushi*, Chigger, Ecology, Thailand

## Abstract

**Background:**

Scrub typhus is an important neglected vector-borne zoonotic disease across the Asia–Pacific region, with an expanding known distribution. The disease ecology is poorly understood, despite the large global burden of disease. The key determinants of high-risk areas of transmission to humans are unknown.

**Methods:**

Small mammals and chiggers were collected over an 18-month period at three sites of differing ecological profiles with high scrub typhus transmission in Chiang Rai Province, northern Thailand. Field samples were identified and tested for *Orientia tsutsugamushi* by real-time PCR. The rates and dynamics of infection were recorded, and positive and negative individuals were mapped over time at the scale of single villages. Ecological analyses were performed to describe the species richness, community structure and interactions between infected and uninfected species and habitats. Generalised linear modelling (GLM) was applied to examine these interactions.

**Results:**

The site with the highest rates of human infection was associated with the highest number of infected chigger pools (41%), individual chiggers (16%), proportion of the known vector species *Leptotrombidium deliense* (71%) and chigger index (151). Chigger species diversity was lowest (Shannon diversity index *H*′: 1.77) and rodent density appeared to be high. There were no consistent discrete foci of infection identified at any of the study sites. The small mammals *Rattus tanezumi* and *Bandicota indica* and the chiggers *L. deliense* and *Walchia kritochaeta* emerged as central nodes in the network analysis. In the GLM, the end of the dry season, and to a lesser extent the end of the wet season, was associated with *O. tsutsugamushi*-infected small mammals and chiggers. A clear positive association was seen between *O. tsutsugamushi-*positive chigger pools and the combination of *O. tsutsugamushi-*positive chigger pools and *O. tsutsugamushi-*positive small mammals with lowland habitats.

**Conclusions:**

These findings begin to reveal some of the factors that may determine high-risk foci of scrub typhus at a fine local scale. Understanding these factors may allow practical public health interventions to reduce disease risk. Further studies are needed in areas with diverse ecology.

**Graphical abstract:**

**Figure:**
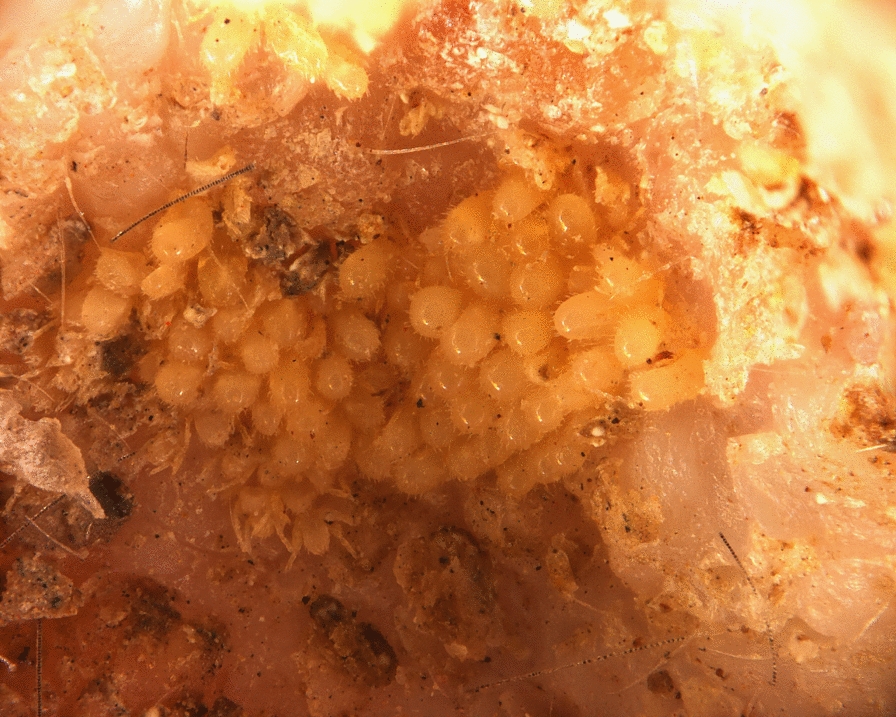

**Supplementary Information:**

The online version contains supplementary material available at 10.1186/s13071-021-05042-4.

## Background

Scrub typhus is a potentially fatal febrile illness with an expanding known distribution, caused by *Orientia tsutsugamushi*, an obligate intracellular bacterium transmitted by the larval stage of trombiculid mites (chiggers) [1]. There are an estimated 1 million cases per year [2], with seroprevalence ranging from 9.3 to 27.9% in Asia [3] and an untreated mortality of approximately 6% [4]. Rural workers and military personnel are typically most at risk, but cases are increasing among city dwellers visiting the countryside [5].

Scrub typhus has a complex and poorly understood ecology, with much existing knowledge dating back to research conducted around World War II, and only in the past two decades has there been a resurgence in research interest in this illness [6]. There are multiple vector chigger species and an even broader range of small mammal and avian hosts [6]. *Orientia tsutsugamushi* is transmitted efficiently transovarially and transstadially, suggesting trombiculid mites act as both vector and host [7, 8]. The pathogen is present in a large variety of habitats, ranging from semi-urban parks and gardens [5] to plantations [9], sandy beaches [10], forest [11] and alpine meadows at 3200 m a.s.l. [12].

Scrub typhus risk appears to be heterogeneously distributed across both small and large geographical scales. Mites exist patchily at the scale of a few metres, possibly due to microclimate requirements [13, 14]. The focalisation of human infection among groups at high risk, such as groups of soldiers, suggests heterogeneity within larger endemic areas [15]. At nation-wide scales disease risk also varies, with the highest disease rates reported, for example, in northern Thailand [16], southwestern Korea [17] and eastern Taiwan [18].

The burden of scrub typhus disease has been rising in Thailand over the past two decades, with 84% of cases reported from northern provinces [16]. Chiang Rai Province has the highest mean number of reported cases per year of 716 (standard deviation: 378), with agricultural workers the most affected. There is a marked seasonality, with cases peaking during the monsoon and harvest periods (July to November).

Ecological factors potentially influencing scrub typhus risk include vector species abundance and richness [19]; rates of *Orientia* chigger infection; small mammal abundance; habitat type including invasive plants [20]; latitude, seasonality and climate [16]; changes in land use [21]; and certain occupations and human behaviours [22, 23].

The interactions between a pathogen, its vector and hosts and the environment are critical to understanding the ecology and evolution of infectious diseases and how to intervene to reduce disease burden [24–26]. A range of statistical measures can be used to analyse these interactions. The number of species present in a given site (species richness) and how species and their abundances are distributed (community structure) can be estimated. Network analysis allows the study of transmission ecology of a pathogen between different vector and host species [27, 28]. Modularity in bipartite and unipartite networks of shared pathogens in vectors, hosts and among habitat types help determine transmission risks [29]. Network centrality scores may provide useful information on the relative importance of a particular element (node) in the network compared with the whole structure [29]. Generalised linear models (GLM) can be applied to test hypotheses associated with the presence of the pathogen in vectors, hosts or habitat types.

Few studies have investigated the ecology of *O. tsutsugamushi* in vectors and hosts over time and at the fine scale of a few square kilometres. Better understanding of these relationships and their interlinkage with human behaviour will be key to understanding the risks of infection and thus strategies to reduce the human disease burden. We investigated chiggers and small mammals over an 18-month period at three sites of high human scrub typhus transmission in Chiang Rai Province, Thailand. Here we report the rates and dynamics of *O. tsutsugamushi* in chiggers and small mammals over time. *Orientia tsutsugamushi*-positive and -negative samples were mapped to identify hotspots and habitat associations. Descriptive methods were used to understand species richness, community structure and interactions (network analysis), with the aim to identify key species and habitats. GLM was used to examine links between *O. tsutsugamushi*-infected chiggers and small mammals with study site-specific variables. We use these links to examine the relationships between trombiculid mites, small mammals, habitat types and the presence of *O. tsutsugamushi*. Our aim was to identify the factors that determine high-risk foci of scrub typhus transmission at the scale of a few square kilometers.

## Methods

### Study site selection and climate description

**Fig. 1:**
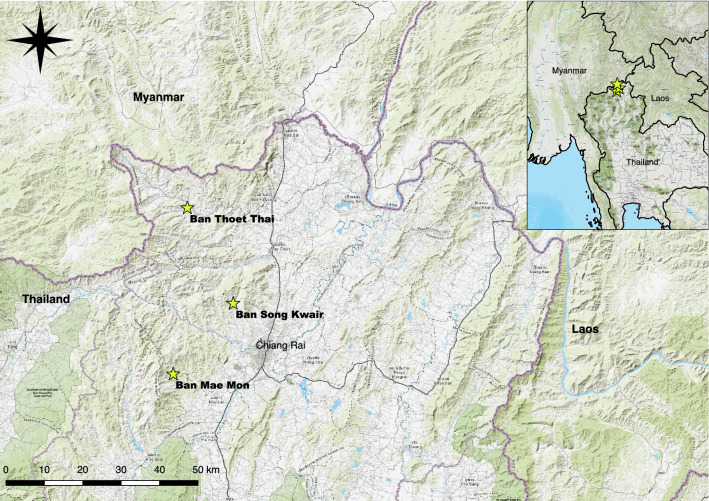
Study site locations, Chiang Rai Province, Thailand

Three villages were selected for detailed investigations of chiggers and small mammals: Ban Thoet Thai (BTT) (20.24°N, 99.64°E), Mae Fahluang district; Ban Song Kwair (BSK) (20.02°N, 99.75°E) and Ban Mae Mon (BMM) (19.85°N, 99.61°E), Mueang district in Chiang Rai Province (Fig. 1). These were selected due to: (i) a high mean annual incidence of scrub typhus in the sub-district (136–395/100,000 population) [16]; (ii) recently identified scrub typhus cases with strong evidence of exposure in peri-village habitats; and (iii) ecological and habitat differences between sites. Detailed descriptions of each village are provided in Additional file 1: Figures S1–S3. The climate is dominated by the southwest monsoon that brings heavy rains from May to October, peaking in August and September. From November to April it is dry, with the occasional shower during March and April. Temperatures peak at over 40 °C in April/May, cool to the low 30 °C range with the onset of the rains and may fall as low as 0 °C at night in higher elevations during December and January.

### Small mammal trapping

Small mammals were trapped alive using locally made wire-mesh traps baited with pieces of corn (*Zea mays*). Approximately 150 traps were placed at intervals of approximately 10 m in lines of 10 traps across each site at dusk and checked daily at dawn for 3 consecutive days. Trap lines were placed to cover the range of habitats present at each study site. Trapping was performed during the cool dry season (January), hot dry season (April), mid-monsoon (August) and end of the monsoon (November).

### Location recording and habitat description

The location of all collected field specimens and traps were recorded using a Garmin Oregon 650 (Garmin International, Olathe, KS, USA) GPS device (accurate to approx. 3 m). A habitat classification scheme was created for all field sites and a basic botanical survey performed. Climate data for the study period was taken from the CLIMATE-DATA.ORG website (https://en.climate-data.org) and included maximum, minimum and mean temperature and rainfall.

### Small mammal identification and processing

Traps containing small mammals were transferred to a mobile laboratory and placed in an airtight box with the addition of cotton wool soaked with 10 ml of the inhalational anaesthetic isoflurane. Animals were only removed from the trap once dead. All animal handling and euthanasia procedures followed international guidelines [30, 31]. All field and mobile laboratory protocols and procedures followed the “Protocols for field and laboratory rodent studies” 2011 guide [32]. This study was approved by the Oxford Tropical Research Ethics Committee (OxTREC 48–15 & 52–14) and by the Kasetsart University Animal Ethics Committee, Bangkok, Thailand (ACKU 62-VTN-010).

Small mammals were weighed in grams and the head and body, tail, hind foot, ear and skull measured in millimetres. Using a combination of morphological features and measurements, small mammals were identified to species level wherever possible following the keys in Chaval [33] and Francis [34]. Prior to harvesting tissues, scissors and forceps were thoroughly washed consecutively in Dettol (chloroxylenol), sterile water and 70% ethanol to prevent cross-contamination. Small pieces (< 50 mg) of lung, liver and spleen were collected into pre-labelled cryotubes and stored on dry ice in the field and subsequently at − 80 °C in the laboratory.

### Chigger removal from small mammals

Both ears were detached as close to the skull as possible and stored in 70% ethanol at 4 °C. The rest of the animal was examined for chiggers, notably the posterior legs, ventral midline and anogenital region. In the laboratory, the ears or skin was examined under a Brunel IMXZ stereo microscope (Brunel Microscopes Ltd., Chippenham, UK) at 15× to 100× magnification. The total number of chiggers per animal were counted. To facilitate collection, a homemade device consisting of a thin stick (3–4 mm in diameter) with a single toothbrush bristle or very fine cactus spine glued to the tip was used to carefully detach the chigger. Chigger index (mean number of chiggers per individual) and chigger infestation (presence of at least 1 chigger on a host animal) were recorded. Representative sets of three chiggers per animal (approx. 5%) were selected based on clusters of different morphological appearance in an attempt to capture the diversity of species present on the host.

### Free-living chigger collection

Two methods were used to collect free-living chiggers: black plates [35, 36] and Berlese funnels (for soil samples) [37]. Black Formica plates (10 × 30 cm) were placed on the ground for 5 min before being examined. A 20 × 20-cm quadrat of the top 5–10 cm of soil and leaf litter was collected into individual plastic bags and transferred to the funnels.

### Chigger identification

Chiggers were placed between two cover slips on a glass slide, and identification to species was performed using a Nikon Eclipse 80i compound microscope (Nikon Corp., Tokyo, Japan) at 400× or 600× magnification. Images were viewed and saved using the Nikon NIS Elements D 4.13.05 software package. Both autofluorescence and bright-field microscopy techniques were used [38]. A scale bar was applied to each image. All morphometric measurements and image manipulation were performed using ImageJ (https://imagej.net/ImageJ). The genus was identified with reference to Nadchatram and Dohany’s 1974 key to Southeast Asian chigger genera [39]. A wide range of taxonomic identification keys were employed to identify chiggers to species level [40–44].

### Chigger and small mammal DNA extraction and PCR

DNA was extracted from individual chiggers, pools of chiggers (approx. 30–50 unidentified individuals, possibly multispecies, from the same host animal) and rodent tissues using the Qiagen Blood and Tissue Kit (Qiagen, Hilden, Germany). Chiggers were rinsed with distilled water and individuals cut through the mid-gut using a sterile 30G needle under a dissecting microscope. Pools were crushed using a sterile polypropylene motorised pestle (Z35991; Sigma-Aldrich, St Louis, MO, USA). Rodent tissues were cut into a small piece (≤ 10 mg of spleen or  ≤ 25 mg of liver or lung), and samples were incubated with proteinase K at 56 °C for 3 h; the rest of the steps followed the manufacturer’s protocol. Chigger samples were eluted in 45 µl and rodent samples in 100 µl of buffer AE (Qiagen). Samples were stored at − 20 °C before performing the PCR assays. Real-time PCR targeting the 47-kDa *O. tsutsugamushi* outer-membrane protein was performed on all samples as previously described [45], except for chigger samples where 5 µl of DNA template was added. PCR assays were run on a CFX96 Touch Real-Time PCR System (Bio-Rad, Hercules, CA, USA).

The association between small mammal organ type and PCR positivity was analysed using a generalised estimating equation (GEE) population-averaged model with the *logit* link function in STATA v15 (StataCorp Inc., College Station, TX, USA).

### Species richness

Species richness was defined as the observed number of chigger or small mammal species found at different study sites. The first-order Jackknife (Jack1) test [46] was used to estimate species richness with the “vegan” v2.5-3 [47] and “BiodiversityR” packages [48] in R freeware (R Foundation for Statistical Computing, Vienna, Austria).

### Species diversity

The Shannon diversity index (*H*′) was used to estimate species diversity as it incorporates both species abundance and evenness and provides a simple summary [49]. This was calculated using the “vegan” [47] and “BiodiversityR” packages in R [48]. Typical values range between 1.5 and 3.5, with the index increasing as the species richness and diversity of the community increase [50].

### Network analysis

The degree of habitat specialisation was estimated using the Paired Difference Index (PDI) [51]. The PDI was calculated for all chigger and small mammal species and for those species testing *O. tsutsugamushi* positive using the “bipartite” package [52]*.* The PDI is measured on a continuous scale from 0 to 1, where a species testing > 0.5 is a habitat specialist and one testing < 0.5, a habitat generalist [51].

Bipartite networks were investigated using either quantitative (i.e. total number of each small mammal or chigger species) or binary (presence/absence) matrices using the “bipartite” package [52]. The following quantitative networks were investigated: (i) small mammal (*n* = 8–9) and chigger species (*n* = 12–21) at each study site; (ii) small mammal species and habitat; (iii) chigger species and habitat. Binary networks were investigated for *O. tsutsugamushi-*positive small mammal species (*n* = 7), *O. tsutsugamushi*-positive chigger species (*n* = 6) and *O. tsutsugamushi*-positive small mammals and chigger species (*n* = 13), with habitat type (*n* = 12) for all sites combined. The function *computeModules* in the R package “bipartite” was used to calculate different sub-communities (modules) within the bipartite network [52]**.**

Bipartite networks were transformed into unipartite networks using the “tnet” package in R [53]. The degree of centrality of a node was measured using the Eigenvector centrality score (EC) with the *evcent* function from the “igraph” package [54]. The transformation of bipartite networks into unipartite networks allows the interaction of “nodes” (species or habitats) in one compartment or sub-community to be analysed [24, 51]. The EC quantitatively measures, on a scale of 0 to 1, the influence of a particular node in the unipartite network being considered. Chiggers or small mammal-habitat networks with individual species EC scores closest to 1 are likely to be most able to occupy multiple habitats and be generalists, whilst those with EC scores closer to 0 are likely to be habitat specialists.

The *cluster_louvain* function in the “igraph” package was used to model unipartite network modularity structure [50].

### Statistical analysis

The relationships between site-specific variables (collection site, low and medium habitat classification type, season, rodent species and chigger genus) were investigated using Goodman and Kruskal’s tau (*τ*) statistic [https://cran.r-project.org/web/packages/GoodmanKruskal/index.html] in R (v0.01). This test measures the strength of associations between categorical data, with values ranging from − 1 (perfectly negative association) to + 1 (perfectly positive association).

Linear discriminant analysis (LDA) was performed using the “MASS” package in R v7.5-53.1 (https://cran.r-project.org/web/packages/MASS/index.html). This visualises the site-specific categories selected and determines whether each site is well characterised by both qualitative and quantitative data describing the sites.

To examine the links between the probability of *O. tsutsugamushi* infection of rodents or pools of chiggers and site-specific variables, we created a generalised linear (mixed) model (GLMM) with logit function using the “arm” package in R. Analyses were performed using the explanatory variables identified by Goodman and Kruskal’s *τ* statistic (low-resolution habitat type, season and elevation). Support for models was assessed using the area under the receiver operating characteristic curve (AUROCC) with the “ROCR” package in R [55].

## Results

### Small mammal trapping

The mean trap success rate was 3.8%. A total of 155 small mammals were trapped, all members of three orders: Rodentia (13 species); Soricomorpha (*Suncus murinus*, the Asian house shrew); and Scandentia (*Tupaia belangeri*, the Northern tree shrew) (Table 1). At each study site, eight to nine species were trapped. At BTT, species that favour rice fields and cultivation were predominant (38/47 of animals trapped, 81%), being either *Bandicota indica* or *Rattus tanezumi*. At BSK, with its high proportion of forest cover, *Rattus andamanensis* was frequently trapped (19/64, 30%), whereas 16/64 (25%) of the small mammals trapped around homes were *Rattus exulans*. At BMM, with its patchwork of habitats, *R. tanezumi* was the most commonly trapped small mammal (20/43, 47%), followed by *Mus cookii* (8/43, 19%) from fallow grassland and cultivated areas.

**Table 1:** PCR testing for *Orientia tsutsugamushi* on spleen, lung and liver tissue (aggregate results) of different small mammal species by site

Study site	Ban Song Kwair (BSK) (%)	Ban Mae Mon (BMM) (%)	Ban Thoet Thai (BTT) (%)	Total (% positive)
*Bandicota indica*	–	–	5/21 (24)	21 (23.8)
*Berylmys berdmorei*	–	–	0/1 (0)	1 (0)
*Berylmys bowersi*	–	1/1 (100)	0/2 (0)	4 (25)
*Callosciurus erythraeus*	0/1 (0)	–	–	1 (0)
*Maxomys surifer*	0/3 (0)	–	–	3 (0)
*Menetes berdmorei*	0/1 (0)	0/3 (0)	–	4 (0)
*Mus caroli*	–	0/1 (0)	0/1 (0)	2 (0)
*Mus cookii*	–	1/8 (13)	1/2 (50)	10 (20)
*Niviventer fulvescens*	–	–	0/1 (0)	2 (0)
*Rattus andamanensis*	6/19 (32)	–	–	19 (31.5)
*Rattus exulans*	3/16 (19)	–	1/2 (50)	18 (22.2)
*Rattus nitidus*	2/8 (25)	0/1 (0)	0/1 (0)	10 (20)
*Rattus* sp.	2/2 (100)	–	–	2 (100)
*Rattus tanezumi*	3/13 (23)	5/20 (25)	8/17 (47)	50 (32)
*Suncus murinus*	–	0/2 (0)	–	2 (0)
*Tupaia glis*	0/1 (0)	0/5 (0)	–	6 (0)
Total	16/64 (25)	7/43 (16)	15/48 (31)	155 (24.5)

### *Orientia tsutsugamushi* PCR of small mammals

Lung, liver and spleen tissues were tested from all trapped animals. At least one tissue tested positive from 38 of the 155 (25%) trapped small mammals (Table 1). Seven species from four genera tested positive. *Rattus tanezumi* and *R. andamanensis* were the species which most frequently tested positive, with 16/50 (32%) and 6/19 (32%)* O. tsutsugamushi*-positive samples, respectively, followed by *R. exulans* (4/18, 22%) and *B. indica* (5/16, 31%). Although sample sizes were small, positive results were also obtained from *Mus cookii* (2/10), *Rattus nitidus* (2/10) and *Berylmys berdmorei* (1/3). Of the three study sites, BTT had the highest proportion of positive animals (15/45, 33%) (BSK: 16/64, 25%; BMM: 7/43, 16%). The total proportion of * O. tsutsugamushi*-positive animals at each field collection varied over time, with a peak of 22/42 (52%) recorded in April 2017.

Of the 155 animals tested, either the spleen or lung was positive in 22 animals and liver samples tested positive in nine animals. In only two of the 38 positive animals were all three organs positive, and in 10 of the 38, two organs were positive. In three cases, the liver was the only positive organ (Additional file 1: Table S1) The mean Cq value for all organs was 37.56 (range 29.5−40.3). The odds ratio (OR) for lung or spleen positivity compared to liver was 2.59 (95% confidence interval [CI] 1.25–5.3; *P* = 0.01).

### Chigger indices and infestation rates

**Fig. 2:**
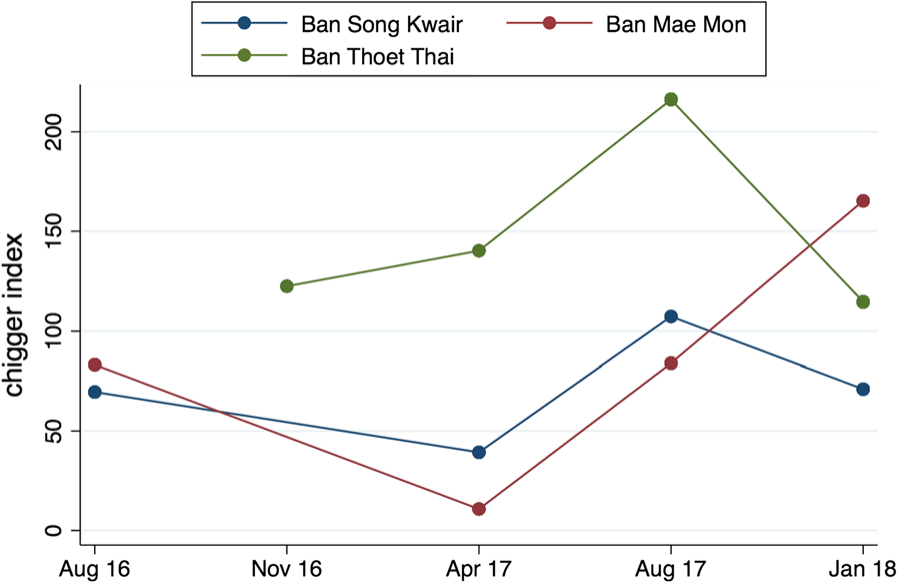
Mean chigger index for all trapped animals at each field visit, by study site

Of the 155 animals trapped, 147 (95%) were infested with chiggers. *Rattus tanezumi* and *B. indica* were heavily infested, with mean chigger indices of 121 and 198, respectively. *Tupaia belangeri* also had a high chigger index (161), albeit from only six individuals. A number of species had low chigger indices; for example, the mean for *Mus* spp. was 27. Mean chigger indices per site were calculated irrespective of the vertebrate species or season of collection. BTT had the highest mean chigger index of 151, followed by BMM with 86 and BSK with 62. Chigger indices were observed to vary over the seasons. Although not entirely consistent between sites, the chigger index peaked during the rainy season in August at BTT and BSK and was lowest at the end of the dry season (April) in BSK and BMM, whilst being lowest at BTT during the mid-dry season in January (Fig. 2).

### Chigger species diversity

A total of 28 confirmed species in 10 genera and two subfamilies were recorded (Table 2). A small number of individuals (12) did not meet all morphometric requirements for species identification in available references and were recorded as ‘sp.’. Chiggers from two additional genera could not be identified due to either a lack of taxonomic keys and/or damage to morphological structures. Two species were recovered only as free-living (*Eutrombicula wichmanni* and *Odontacarus audyi*). Three other acarine families, Cheyletidae, Laelapidae and Sarcoptidae (*Notoedres* sp.), were removed from rodents. Chigger species richness varied between the different study sites with 25 at BMM, 23 at BSK and just 15 at BTT. Species richness did not vary appreciably at different times of the year. Taking into consideration only the two recognised human vector species, *Leptotrombidium deliense* and *Leptotrombidium imphalum*, the proportion was highest at BTT (116/163, 71%), followed by BSK (87/197, 44%) and BMM (42/156, 27%). *Walchia* spp. and *Ascoschoengastia indica* were the other major species recorded. *Leptotrombidium deliense* was recorded from 11 small mammal species and *L. imphalum* from four small mammal species. *Rattus* spp. and *B. indica* combined hosted 85% of *L. deliense* and 99% of *L. imphalum*.

**Table 2:** *Orientia tsutsugamushi* PCR testing on individual chiggers and other acarines

Study site	Ban Song Kwair (BSK) (%)		Ban Mae Mon (BMM) (%)		Ban Thoet Thai (BTT) (%)		Total (% PCR positive)
*Ascoschoengastia indica*	0/39 (0)		0/10 (0)		0/1 (0)		50 (0)
*Ascoschoengastia* sp.	–		0/1 (0)		–		1 (0)
*Cheyletus* sp.^a^	0/2 (0)		0/1 (0)		–		3 (0)
*Eutrombicula wichmanni*	0/1 (0)		–		–		1 (0)
*Gahrliepia* sp.	0/3 (0)		0/6 (0)		0/1 (0)		10 (0)
***Gahrliepia xiaowoi***^b^	**0/1 (0)**		**1/6 (17)**		–		**7 (14.2)**
*Helenicula kohlsi*	0/3 (0)		–		–		3 (0)
*Helenicula naresuani*	0/6 (0)		0/4 (0)		–		10 (0)
*Helenicula pilosa*	–		–		0/2 (0)		2 (0)
*Helenicula scanloni*	–		0/11 (0)		–		11 (0)
*Helenicula* sp.	0/1 (0)		–		–		1 (0)
*Laelapidae*^a^	0/2 (0)		0/1 (0)		0/4 (0)		7 (0)
*Leptotrombidium arvinum*	–		0/1 (0)		–		1 (0)
***Leptotrombidium deliense***	**3/79 (3.8)**		**0/36 (0)**		**13/33 (39)**		**148 (10.8)**
*Leptotrombidium elisbergi*	–		0/2 (0)		–		2 (0)
*Leptotrombidium harrisoni*	–		0/4 (0)		–		4 (0)
***Leptotrombidium imphalum***	**0/8 (0)**		**0/6 (0)**		**4/80 (5)**		**94 (4.2)**
*Leptotrombidium* sp.	0/2 (0)		0/1 (0)		0/2 (0)		5 (0)
*Leptotrombidium turdicola*	0/1 (0)		0/13 (0)		–		14 (0)
*Notoedres* sp.^a^	–		–		0/1 (0)		1 (0)
*Odontacarus audyi*	0/2 (0)		–		–		2 (0)
*Schoengastiella ligula*	0/8 (0)		–		–		8 (0)
***Schoutedenichia *sp.** ^b^	**1/1 (100)**		–		**1/1 (100)**		**2 (100)**
*Susa traubi*	0/1 (0)		–		–		1 (0)
*Trombiculindus* sp.	0/1 (0)		0/17 (0)		–		18 (0)
*Trombiculindus paniculatum*	–		0/1 (0)		–		1 (0)
***Trombiculindus variaculum***^b^	–		–		**1/1 (100)**		**1 (100)**
*Walchia alpestris*	–		0/4 (0)		–		4 (0)
*Walchia dismina*	–		0/2 (0)		–		2 (0)
*Walchia disparunguis*	–		0/3 (0)		–		3 (0)
*W. disparunguis disparunguis*	0/4 (0)		0/2 (0)		–		6 (0)
*Walchia ewingi lupella*	–		–		0/2 (0)		2 (0)
***Walchia kritochaeta***^b^	**1/23 (4.3)**		–		**6/22 (27)**		**45 (15.5)**
*Walchia micropelta*^b^	0/1 (0)		0/18 (0)		0/7 (0)		26 (0)
*Walchia minuscuta*	–		0/1 (0)		–		1 (0)
*Walchia rustica*	0/7 (0)		–		–		7 (0)
*Walchia* sp.	–		–		0/2 (0)		2 (0)
*Walchia turmalis*	–		0/4 (0)		–		4 (0)
*Walchia ventralis*	–		0/2 (0)		–		2 (0)
*Walchiella* sp.	0/1 (0)		–		–		1 (0)
**Total**	**5/197 (2.5)**		**1/157 (0/6)**		**25/159 (15.7)**		**513 (6)**

Species with positive PCR results are indicated in bold

^a^Indicates other Acari removed from small mammals by site

^b^Indicates species not previously reported as *O. tsutsugamushi* positive

### *Orientia tsutsugamushi* PCR of individual chiggers, chigger pools and free-living chiggers

In total, 513 individual chiggers removed from 147 small mammals were tested (Table 2), with 31 (6%) found to be *O. tsutsugamushi* positive from six chigger species. Three species comprised 90% of all positive chiggers: *L. deliense* (16/148, 11%), *L. imphalum* (4/94, 4.3%) and *Walchia kritochaeta* (7/45, 16%). The vast majority of *O. tsutsugamushi-*positive chiggers from small mammals were from BTT (25/31, 81%), with only three from BSK and none from BMM. Individual chiggers contained between 11 to 2850 genome copies/µl of *O. tsutsugamushi* by quantitative PCR (qPCR).

**Fig. 3:**
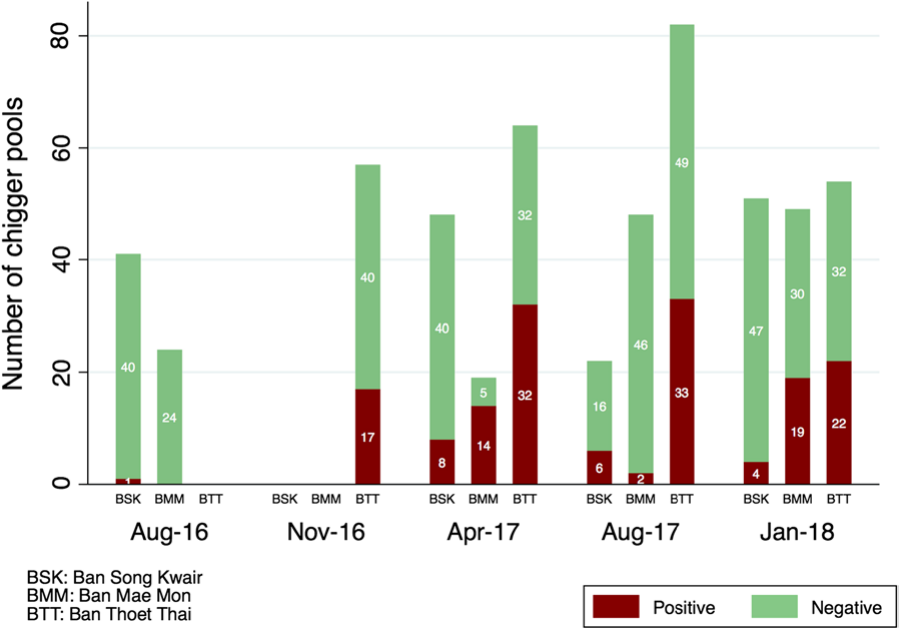
Proportion of chigger pools that tested positive for *Orientia tsutsugamushi* by PCR according to time of year collected, by study site

In total, 559 pools of chiggers (exact species composition undetermined) were prepared and tested for *O. tsutsugamushi*, consisting of a total of 14,826 chiggers. The mean pool size was 26 (range: 10–63) chiggers. Chiggers were pooled by individual host animal and the pools were composed of either single or mixed species. *Rattus tanezumi* and *B. indica* were the most numerous and heavily infested rodent species and accounted for the largest proportion of positive pools (42 and 25%, respectively). In total 158 (28%) of chigger pools were positive for *O. tsutsugamushi*. Nine small mammal species had at least one positive pool; however, *R. tanezumi* and *B. indica* comprised 80% of positives. BTT had consistently the highest proportion of positive samples (104/257, 41% overall) through different seasons. The proportion of positive pools ranged from 27% during the rainy season of August 2017 to 41% at the end of the dry season in April 2017 (Fig. 3). At BMM and BSK, 35/140 (25%) and 19/162 (12%) of chigger pools were *O. tsutsugamushi *positive.

Free-living chiggers were collected from a variety of habitats at all sites. Seven chigger species (32 individuals) were collected from BSK, of which 21 were *L. deliense* and four *L. imphalum.* Eight species (34 individuals) were collected from BMM, predominantly *Gahrliepia* spp. and *Trombiculindus* spp. and three *L. deliense*. At BTT, four *L. imphalum* were collected. PCR testing for *O. tsutsugamushi* was performed on 70 chiggers, and four were positive (5.7%) (Table 3).

**Table 3:** PCR testing for *Orientia tsutsugamushi* on individual free-living chiggers

Chigger species	PCR negative	PCR positive (%)
*Eutrombicula wichmanni*	1	0
*Gahrliepia* sp.	7	0
*Gahrliepia xiaowoi*	6	**1 (14.2)**
*Leptotrombidium deliense*	22	**2 (8.3)**
*Leptotrombidium imphalum*	7	**1 (12.5)**
*Leptotrombidium turdicola*	4	0
*Odontacarus audyi*	2	0
*Trombiculindus* sp.	16	0
*Walchia turmalis*	1	0
Total	66	**4 (5.7)**

Positive PCR results are indicated in bold

### Habitat description

A habitat classification scheme of low and medium resolution was created based on the study site habitats (Additional file 1: Table S2). Twenty-one of the most frequently encountered plant species were identified (Additional file 1: Table S3). The majority of these (13 species) were grasses and bamboos (Graminae). Wild sugarcane *Saccharum spontaneum*, three species of invasive African grasses of the genus *Pennisetum*, *Imperata cylindrica* (a common grass of East Asia) and two bamboo species dominated. *Ageratum conyzoides* and *Bidens pilosa* were very widespread invasive Compositae.

### Spatial distribution of *O. tsutsugamushi*-positive and -negative chiggers and small mammals

The location of all trap positions of *O. tsutsugamushi*-positive and -negative chiggers and small mammals at each study site over the course of the study showed no clear evidence of persistently positive or negative foci of infection (Additional file 1: Figures S1–S3).

### Species richness and diversity of chiggers on small mammal hosts

**Fig. 4:**
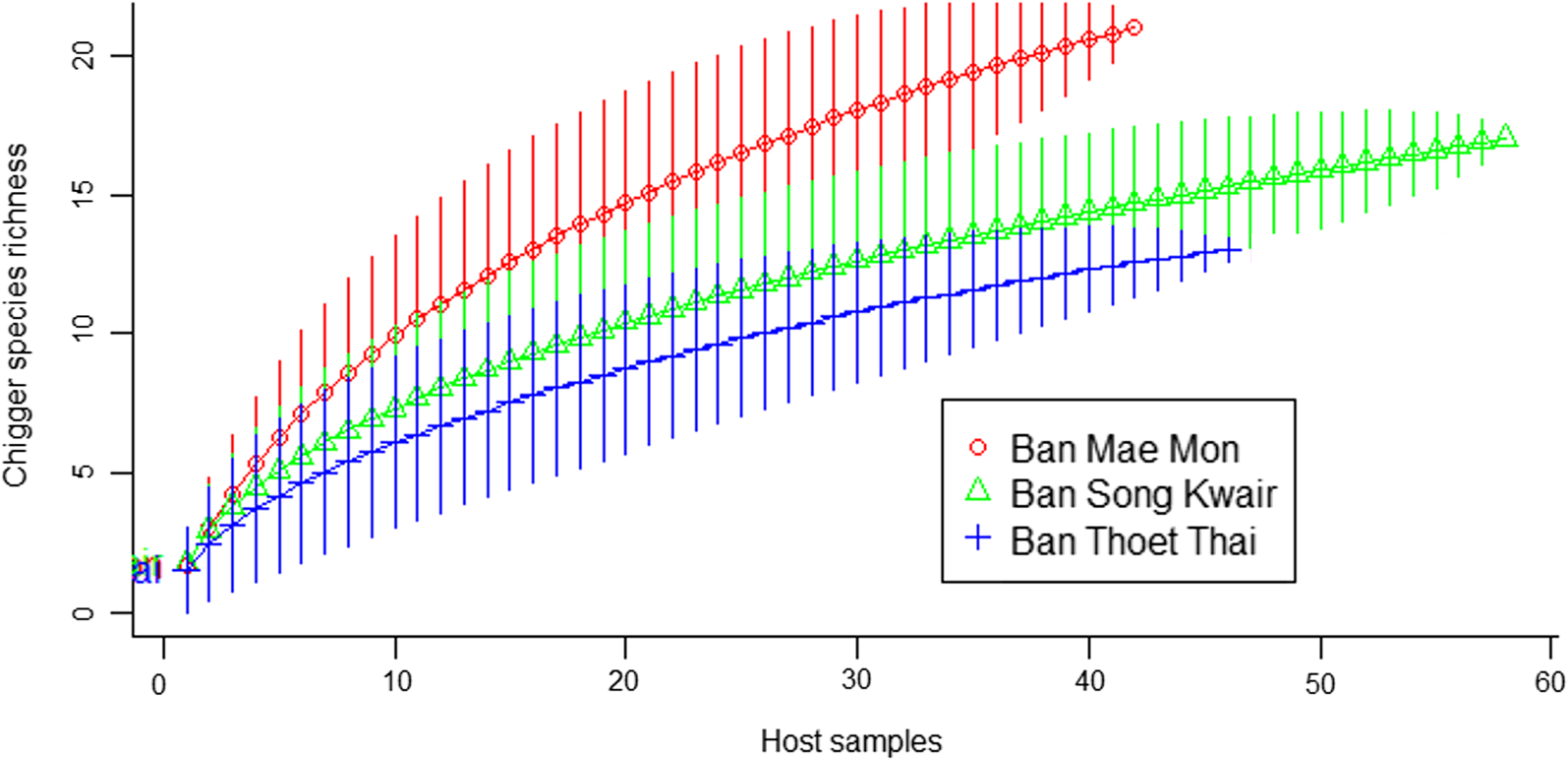
Rarefaction curves for chigger species richness at each study site. The curves represent mean chigger species richness, and the bars indicate the standard deviation

Estimates of chigger species richness on small mammals at each of the study sites were made. The highest species richness was observed for BMM (Jack1 = 29.8), followed by BSK (Jack1 = 24.8) and BTT (Jack1 = 17.9). Rarefaction curves for each site (Fig. 4) suggest that BTT was the site closest to sampling saturation.

The Shannon index was measured for the three main sites to estimate chigger diversity on small mammals. At BMM, *H*′ was 2.58 (95% CI 2.46–2.71); at BSK, *H*′ was relatively lower at 2.04 (95% CI 1.94–2.14). Diversity was lowest at BTT with *H*′ = 1.77 (95% CI 1.69–1.85).

### Small mammal species and chigger network analysis

**Fig. 5:**
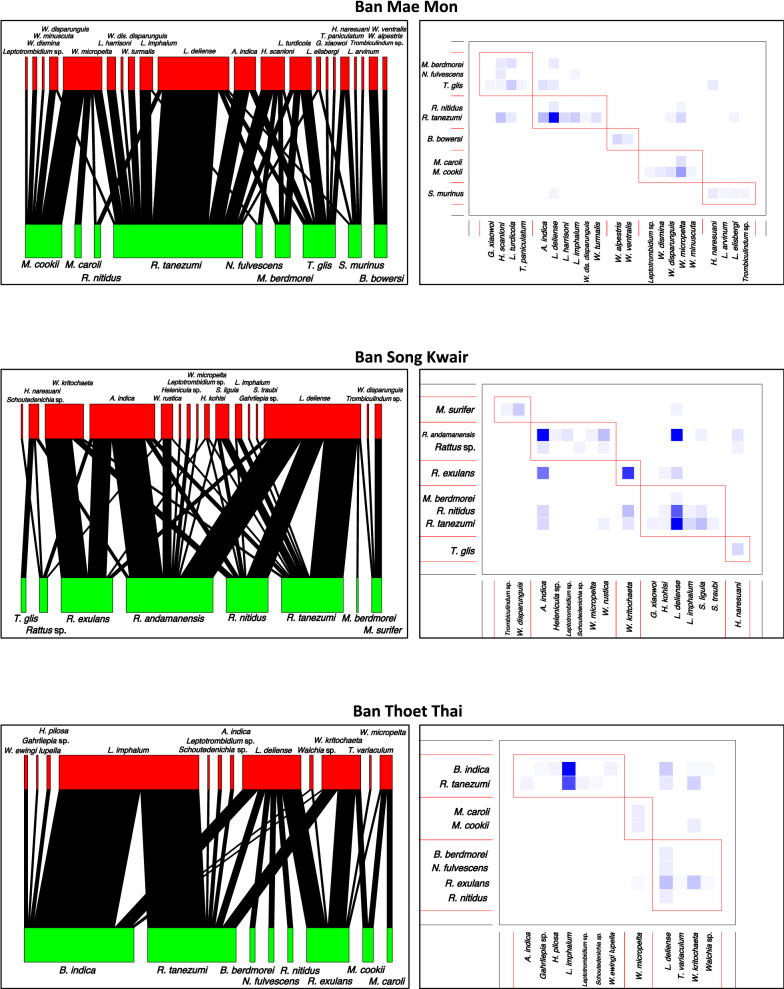
Quantitative bipartite network figures (left column) and sub-community modules (right column) for small mammal-chigger interactions. The degree of shading of the module blue boxes reflects the size of the small mammal–chigger species pair interaction

**Fig. 6:**
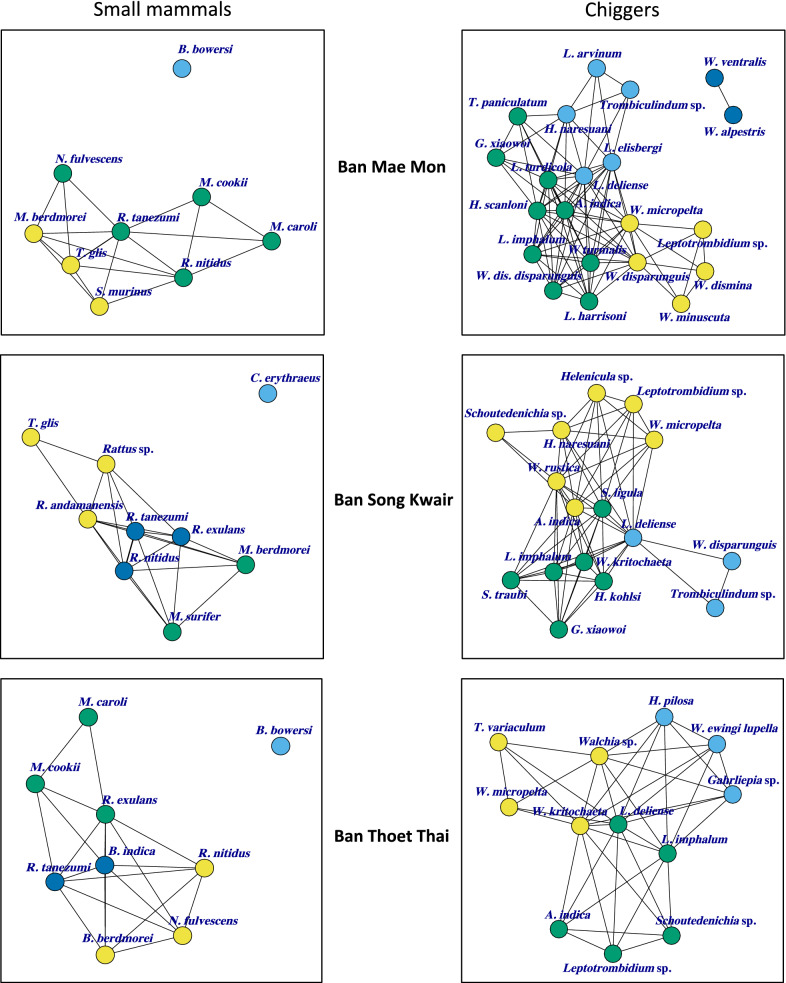
Unipartite network models for small mammal–chigger interactions. The modules are shown by different colours. The most centrally located node has an Eigenvector score closest to 1

Habitat diversity and chigger species richness were lowest at BTT. Sub-community analysis grouped *B. indica* and *R. tanezumi* most closely with *L. imphalum. Bandicota indica* was central (EC = 1) among host species. Despite its abundance, *L. imphalum* was not central (EC = 0.46), with *W. kritochatea* with an EC = 1 and *L. deliense* an EC = 0.72. *Rattus tanezumi* and *L. deliense* were central in the unipartite network (EC = 1). Sub-community analysis at BSK, an area predominantly consisting of secondary forest, grouped *L. deliense* and *L. imphalum* with *R. tanezumi*, *R. nitidus* and *Menetes berdmorei*. *Rattus andamanensis* was also closely associated with *L. deliense.* The EC scores were 1 for *R. tanezumi* and *A. indica*, 0.94 for *L. deliense* and 0.77 for *W. kritochaeta* (Figs. 5, 6).

### *Orientia tsutsugamushi*-positive small mammal and chigger network analysis

**Fig. 7:**
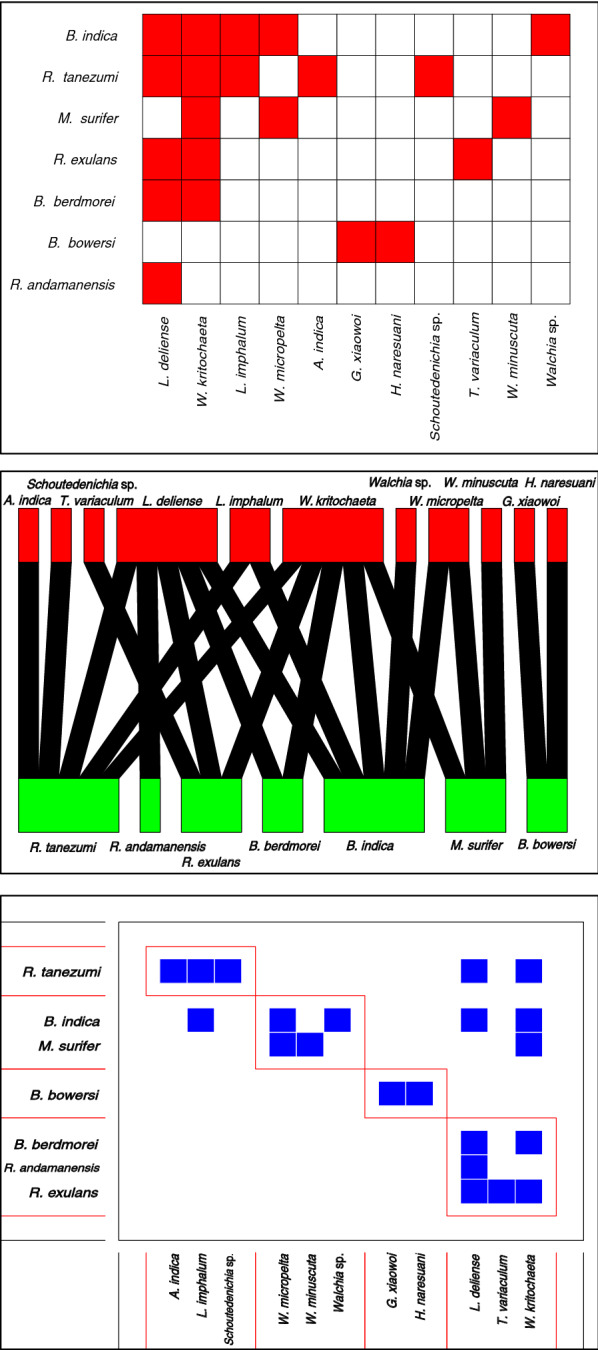
Non-quantitative nestedness matrix, bipartitie network and sub-community modules for chigger–small mammal interactions where either species tested *O. tsutsugamushi* positive

A binary network analysis was used to analyse the interactions between *O. tsutsugamushi*-positive chiggers and their host species. Sub-community analysis revealed *L. imphalum* to be associated with *R. tanezumi*, while *B. indica* and *Maxomys surifer* were grouped with *Walchia micropelta* and *Walchia minuscuta. Leptotrombidium deliense* and *W. kritochaeta* were grouped with *R. andamanensis*, *R. exulans* and *B. berdmorei.* Centrality scores placed *B. indica* and *W. kritochaeta* centrally (EC = 1), with *L. deliense* at 0.93, *L. imphalum* at 0.42, *R. tanezumi* at 0.83 and *R. andamanensis* at only 0.21 (Fig. 7).

### *Orientia tsutsugamushi*-positive chiggers/small mammals and habitat network analysis

A binary matrix was also used to analyse the interaction between *O. tsutsugamushi*-infected vectors/hosts with habitat types. *Leptotrombidium deliense* and *R. tanezumi* were identified more clearly as habitat generalists, with a PDI of 0.33 and 0.42, respectively. Paddy field/riverbank (EC = 1), corn field (0.91) and mixed secondary forest (0.83) were most central in the unipartite network (Additional file 1: Figure S4).

### Statistical analysis

Goodman and Kruskal’s *τ* showed good positive association between low-resolution habitat type and season, but not medium-resolution habitat type (values > 0.6) (Additional file 1: Figure S5). As a result, GLMM was performed with three variables: low-resolution habitat type, season and elevation.

**Fig. 8:**
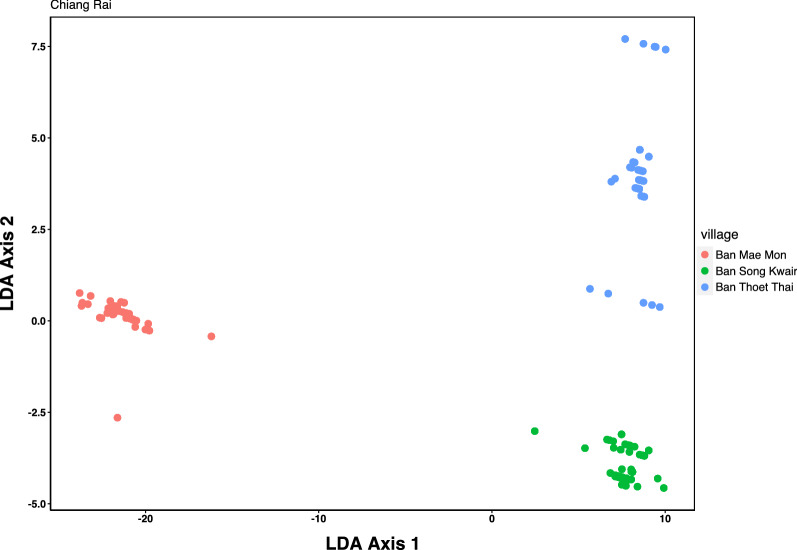
LDA for site variables (elevation, low and medium resolution habitat type and season) at each study site. Elevation is given in meters above sea level. Abbreviations: LDA, Linear discriminant analysis

The LDA showed that each study site displayed well-characterised environmental variables, although BTT had greatest variability among these characteristics, reflected by the spread across axis 2 (Fig. 8).

**Fig. 9:**
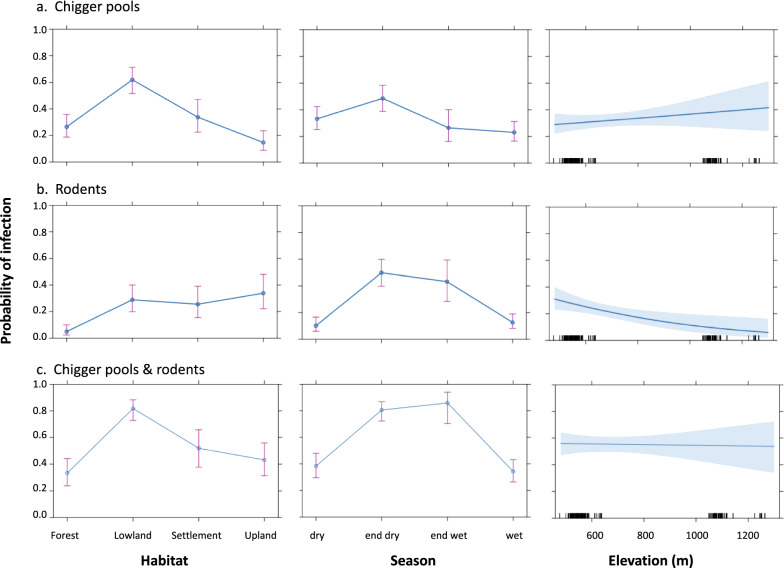
Generalised linear (mixed) model analysis of the effect of low-resolution habitat type, season and elevation on the probability of infection with *O. tsutsugamushi* (PCR positive) in chigger pools (**a**), rodents (**b**) and combined chigger pools and rodents (**c**)

The GLMM analysis of *O. tsutsugamushi*-positive rodents showed a slightly stronger association with upland habitats, lowland and settlements compared to forested habitats (all *P*-values < 0.001). However, *O. tsutsugamushi* positivity showed a modest decreasing association with rising elevation (*P*-value < 0.001). Changing seasons (end of the dry and end of the wet) were most strongly associated with *O. tsutsugamushi* infection (all *P*-values < 0.001). The model was well supported (pseudo-*R*^2^ = 0.23, null deviance = 542,* df* = 456; Chi-squared goodness of fit = 424.0 with a *P*-value = 0.96) with an AUC of 0.79 (95% CI: 0.74–0.83) (Fig. 9).

The GLMM analysis of *O. tsutsugamushi*-positive chigger pools showed a clear positive association with lowland habitats (*P*-value < 0.001) and a negative association with upland habitats (*P-*value < 0.05) compared to settlement and forested habitats, and no signification association with elevation (*P*-value = 0.31). The end of the dry season was most closely associated with infected chigger pools (*P*-value < 0.001). The model was well supported (pseudo-*R*^2^ = 0.14, null deviance = 589, *df* = 456; Chi-squared goodness of fit = 468.7 with a *P*-value = 0.13) with an AUC of 0.68 (95% CI: 0.63–0.74) (Fig. 9).

A GLMM analysis of *O. tsutsugamushi* combining positive rodents and positive chigger pools for each individual rodent showed a strong association with lowland habitats (*P*-value < 0.001) and a slight association with settlement habitats (*P*-value = 0.04), but no association with elevation (*P*-value = 0.87). Changing seasons (end of the dry and end of the wet) were strongly associated with *O. tsutsugamushi* infection. The model was well supported (pseudo-*R*^2^ =  0.29, null deviance = 633,* df* = 456; Chi-squared goodness of fit = 419.5 with a *P-*value = 0.54) with an AUC of 0.78 (95% CI: 0.73–0.82) (Fig. 9).

A GLMM could not be performed for individual *O. tsutsugamushi*-positive chiggers due to low numbers.

## Discussion

Very little is currently understood about the factors that determine an area to be at high risk for scrub typhus and what the size and temporal dynamics of such a site might be. By investigating the disease ecology of scrub typhus, we can advance our understanding of these factors, with the ultimate aim of developing important practical public health interventions.

Three sites of high human scrub typhus transmission with different environmental and ecological profiles were selected and studied over an 18-month period. The three sites differed in ecological profiles, with altitudes ranging from 550 to 1200 m a.s.l., composed of varying habitats (Additional file 1: Figures S1–S3). Ban Thoet Thai (BTT) had the highest frequency of *O. tsutsugamushi* in chiggers (individuals and pools removed from small mammals and free-living individuals) and small mammals and is also one of the sub-districts with the highest human scrub typhus incidence in Thailand [16]. A total of 41% of chigger pools tested positive for *O. tsutsugamushi*, compared to 25% at BMM and 12% at BSK. Similarly, 16% of individual chiggers at BTT tested positive, with < 1% at BSK and BMM testing positive. BTT also had the highest mean chigger index (151) and the lowest chigger species diversity (*H*′= 1.77). BTT had the highest proportion of the two major known vector species, *L. imphalum* and *L. deliense* (71%).

The chigger index has been widely regarded as a proxy for scrub typhus risk [56–58]. In more northern latitudes, chigger indices fall markedly over the winter, as does human scrub typhus incidence, whilst in tropical latitudes chigger numbers fall during the dry season when conditions are less suitable for the chigger life-cycle to be completed [6]. In this study, chigger indices were similarly lower during the early and late dry season compared to the wet season, corresponding to the regional temporal pattern of human scrub typhus (Fig. 2). A higher chigger index may correspond to a greater risk of infection with *O. tsutsugamushi*. However, particularly at BTT, the proportion of *O. tsutsugamushi*-positive chigger pools (perhaps the best available proxy for scrub typhus risk) ranged from 41 and 50% in the dry season and from 30 to 37% in the wet season. This does not correlate with human incidence [16]. This persistence of PCR-positive vectors regardless of season at that same location, not been previously reported, suggests that the mite life-cycle continues throughout the year at this location and that humans are accidental hosts. High-risk occupational activities (farming/harvesting) bring humans, rodents and chiggers together.

The bipartite networks illustrate clearly that certain species of chigger have only a few host species (*L. imphalum* found on *B. indica* and *R. tanezumi*), while others, such as *L. deliense*, have a wide host species range (Fig. 5). *R. tanezumi*, *B. indica* (rodents) and *L. deliense*, *W. kritochaeta* (chiggers) emerged as central nodes in the network analysis (Fig. 6). In the few other studies that have been conducted on small mammal–ectoparasite interactions in Southeast Asia, *B. indica*, *R. tanezumi*, *Bandicota savilei*, *R. exulans* and *L. deliense* were consistently centrally placed in the network analyses [20, 26]. The central role of *W. kritochaeta* and the identification of 7 of 31 (23%) *O. tsutsugamushi*-positive individuals suggest that *Walchia* spp. may play a more important role in scrub typhus than currently believed, possibly as an intrazootic vector. In this analysis, the paddy field/riverbank habitat was most centrally placed with an EC of 1.

The LDA shows that the three study sites were well discriminated based on the variables elevation, low and medium resolution and season (Fig. 8). An association with the end of the dry season, and to a lesser extent, to the end of the wet season was seen for both *O. tsutsugamushi*-positive rodents and pools of chiggers in the GLMM, suggesting an importance of seasonal transition. Neither analysis showed an association of *O. tsutsugamushi* positivity in chiggers or rodents with the peak wet season, the period with the highest incidence of scrub typhus cases. The end of the dry season sees the first heavy rain showers, which may stimulate a rise in (*O. tsutsugamushi*-infected) chigger density. Lower elevation appeared to be associated with a higher number of infected rodents, although a slight increase in infected chiggers was seen with higher elevations. Lowland habitat was clearly associated with infected chiggers, while the habitat association with rodents was less marked. Forest and upland were the habitats least associated with infected chiggers. These results were reinforced when the analysis was performed on both individual and pools of infected chiggers. Lowland habitat and settlement habitats were found to be associated with *O. tsutsugamushi* infection, occurring mostly at the end of the dry and the wet seasons (Fig. 9). Forest habitat was poorly associated with *O. tsutsugamushi* infection in all the analyses. These results are consistent with those of previous studies which suggest that disease risk is associated with ecotones [6].

We created a medium-resolution habitat classification system to describe the three study sites. No standardised habitat classification system currently exists, and at the scale of these study sites—typical daily exposure areas for at-risk humans—a complex mosaic of habitats is present. Small mammals are likely to visit several habitats during foraging, although little data are available on home ranges. The GLMM rejected the medium-resolution habitat association, probably due to too many and inconsistent variables between sites. A simplified and applicable habitat classification scheme is needed for future studies.

This study mapped the spatial distribution of *O. tsutsugamushi*-positive and -negative chiggers and their small mammal hosts at multiple time points across seasons at a high-resolution scale of just a few square kilometres in an endemic area. At this scale there were no consistently positive areas to suggest high-risk foci. These results are not able to take small mammal home ranges into account, which remain poorly known. A recent study reported that infected chiggers and rodents were associated with certain sites and with grasslands, forest areas and dense forest edges at a similar scale [59]. However, the dynamics of infected and uninfected chiggers and rodents over time and across different seasons and sites were not reported. The description of “mite islands” in World War II (WW2) as areas of high risk for scrub typhus may have been overemphasised in subsequent literature. The scenario encountered in WW2 was that of immunologically naïve soldiers being exposed in large numbers at high human density and in very poor living conditions in small foci. This is quite different to modern-day scenarios where rural workers are exposed regularly.

There are many challenges to investigating sites of high disease transmission. It is usually impossible to be certain where a person was actually bitten to acquire scrub typhus. It is very resource intensive to attempt small mammal trapping and searches for free-living chiggers over large areas. Trapping rodents is not always successful due to population fluctuations as a consequence of climate patterns, food availability and pressure from hunting by humans [60]. As a result of efficient transovarial transmission and given that chiggers probably only feed once in their life-cycle, *O. tsutsugamushi*-positive free-living chiggers implicate the species as a vector of the disease. In addition to *L. deliense* and *L. imphalum*, *Gahrliepia* *xiaowoi*, not a known vector, tested positive for *O. tsutsugamushi*. Thus, collecting free-living chiggers is crucial to understanding the disease ecology. Improvements in mite identification using autofluorescence allowing matched morpho- and genotyping and simultaneous pathogen detection will advance this knowledge [38]. Relatively few studies have collected free-living chiggers; however, studies from Malaysia, Japan and Australia report that as many as 10,000 larvae were collected from certain hotspots [13, 61, 62]. The distribution and location of free-living chiggers in the environment and their determinants remain poorly understood.

The spleen and lung of small mammals were significantly more likely to be *O. tsutsugamushi* positive than the liver. Studies using the mouse model suggest that the lungs are an important site of pathology [63]. Only two studies have compared PCR detection rates in different organs, and both assessed the liver, spleen and kidney [64, 65]. The spleen proved to be the organ most frequently positive, but kidney alone was positive in three rodents. This result underlines the importance of testing multiple tissue types.

The results of qPCR assays suggest that individual chiggers contain between 11 to 2850 genome copies/µl of *O. tsutsugamushi*. Two recent studies in Thailand document qPCR results on individual (wild) chiggers, with a range of 13.8 to 2251.6 copies/µl [59, 66].

There have been 32 published scrub typhus investigations in vectors and hosts in Thailand since 1952 [6, 19, 59, 67–69]. Ten studies report testing vectors and/or hosts for *O. tsutsugamushi* using molecular methods, including PCR and 16S ribosomal RNA amplicon sequencing from several sites around Thailand [19, 59, 64, 66, 68–73]. Rodent tissue *O. tsutsugamushi* PCR positivity ranged from 0 [69] to 22% [71], and chigger positivity ranged from 0 [69] to 15.6% [71], with a mean of approximately 5% positivity. Khuntirat et al. [64] reported *O. tsutsugamushi* PCR positivity in two of 31 individual chiggers and 2–10% rodent tissues based on identification of the *O. tsutsugamushi* 56-kDa fragment, from 2 villages in Chiang Rai Province. Linsuwanon et al. [59] recorded PCR positivity in tissues of 5/91 (5.5%) rodents and in 67/1415 (4.7%) chiggers in a small area in the east of Chiang Rai Province; in this study, *O. tsutsugamushi*-positive chiggers were predominantly *L. deliense *[59]*.*

Only two previous studies investigating free-living chiggers for *Orientia* sp. have been conducted in Thailand, published in 1966 [74] and 1981 [75]. These report that 6/17 (35%) *L. deliense* pools were positive by xenodiagnosis and that 146/3764 (3.9%) chiggers were positive by direct immunofluorescence antibody testing, respectively. False positives are a concern with IFA [76].

The PCR positivity rates of both small mammal and individual chiggers were higher in this study than reported in other studies, particularly at BTT. We report *O. tsutsugamushi* PCR-positive chiggers removed from rodents for the first time of *Schoutedenichia* sp., *Trombiculindus variaculum* and *W. kritochaeta*; and in a free-living *Gahrliepia* *xiaowoi.*

There has been very little research into scrub typhus ecology in recent decades. Such studies in Thailand report differing associations with habitat. *Orientia tsutsugamushi* infection in rodents was associated with heterogeneous forest habitats [68]. *Orientia tsutsugamushi* infection in chiggers in eastern Chiang Rai Province was associated with grassland and forest ecotones, although the strength of the association was not reported [59]. At the sub-district level in Chiang Rai Province, the number of scrub typhus cases was associated with ‘forest open cover’ (%) (OR 1.56; 95% CI 1.01–2.41), but not associated with forest (OR 0.81; 95% CI 0.77–0.86) [16]. In this study, forest cover and upland habitats were least associated with *O. tsutsugamushi*-infected chiggers.

Increased humidity has been associated with a greater relative risk of infection in Thailand [77]. At the scale of 10-km^2^ sites in 11 provinces in Thailand, chigger species richness was positively associated with human scrub typhus incidence at the sub-district scale [19]. This is the converse to what was identified in this study. In Bangkok Metropolitan parks, chigger abundance was associated with proximity to open fields, although no forest cover was present in this study area [69]. No *O. tsutsugamushi*-positive chiggers or rodents were found in this study, and *L. deliense* was present at some sites, forming 20% of chiggers identified. This is a considerably lower proportion than that found in the present study (up to 70%)). The proportion of *O. tsutsugamushi*-infected chiggers was highest at the end of the dry season (April) in the present study. At a nearby site in the east of Chiang Rai Province [59], abundance of infected chiggers was highest in December, although that study had few time points.

These contradictions reflect the varying scales, time periods and measures of risk (chiggers, rodents and humans) employed in the different studies. Habitat complexity and ecotones appear to be important factors, although the reasons for this remain unknown. It is clear that no single factor dominates in explaining scrub typhus risk and that this varies geographically. It is likely that human behaviour is a more critical risk factor for acquiring scrub typhus than is widely credited in the literature. Chiggers probably do not bite humans without sufficient opportunity to attach. In South East Asia, farming behaviour is very seasonal, with less field activity from January to April. During the concentrated planting and harvesting periods, many rural people are involved and long periods of time are spent in the fields. These periods coincide with peak human scrub typhus incidence [16]. Infected chiggers are present throughout the year, but the number of human cases rise and fall, most likely corresponding to the risk of exposure.

There are several weaknesses inherent in this field study. Firstly, the sampling effort was limited to four investigations at each site in Thailand over the course of 18 months. Of the total sampling visits, on two occasions not all sites were sampled. Sampling success (rodent capture rates) also varied between sites and visits. Rarefaction curves suggest sample size could have been improved (Fig. 4). Secondly, only three chiggers per host (approx. 5% of total) were identified to species level. Chaisiri et al. reported that 16% of small mammals were infested by > 3 chigger species [19]; thus it is likely that the overall diversity was underreported in the present study. However, identification of all chiggers attached to five randomly selected hosts suggested that the method successfully captured overall diversity. Thirdly, the medium resolution of classification of habitat types was too complex and inconsistent between sites, resulting in failure of GLMM. This should be urgently resolved for future studies.

## Conclusion

This study is a first step towards identifying important factors associated with high-risk transmission sites and underlines the importance of conducting further detailed ecological investigations in other regions, particularly where scrub typhus has recently been recognised, to determine whether these factors are generalisable.

## Supplementary Information

**Additional file 1: Table S1.** *Orientia tsutsugamushi* PCR positivity by organ type and small mammal species. **Table S2.** Habitat classification scheme. **Table S3.** List of the most common plants identified at the study sites (1 = Ban Thoet Thai, 2 = Ban Song Kwair, 3 = Ban Mae Mon). **Figure S1.** Spatial distribution over time of all trap positions and *O. tsutsugamushi*-positive chigger pools, individual chiggers and small mammals for Ban Thoet Thai. This is the major town of the sub-district of Mae Fahluang district. Population: approx. 5,000; elevation: 550 m a.s.l.; mixed hill tribe ethnicities. Study site is located along a small river, encompassing rice fields, vegetable/fruit gardens, patches of fallow areas, degraded secondary forest and a few homes. **Figure S2.** Spatial distribution over time of all trap positions and *O. tsutsugamushi*-positive chigger pools, individual chiggers and small mammals for Ban Song Kwair. Home of human case is shown with star. This is an isolated small village in Mae Yao sub-district. Population: approx. 150; elevation: 650 m a.s.l.; Akha hilltribe village, predominantly with traditional wooden stilt homes. Study site is located beside a fast-flowing stream, at the head of the valley; it is surrounded by mixed secondary forest, teak plantation, fallow areas and bamboo groves, and there are patches of dry rice, corn and pineapple plantations. **Figure S3.** Spatial distribution over time of all trap positions and *O. tsutsugamushi*-positive chigger pools, individual chiggers and small mammals for Ban Mae Mon. Home of human case shown with star. The village is located in Huay Chomphu sub-district. Population: approx. 1500; elevation: 1200 m a.s.l.; mixed village of Akha and Lisu hill tribe people, predominantly concrete constructed homes. The village located on the steep slope of a ridge, surrounded by coffee, fruit and vegetable plantations, fallow areas and a small secondary forest. **Figure S4.** Non-quantitative nestedness matrix, bipartitie network, sub-community modules and unipartite network models for *O. tsutsugamushi*-positive chigger/small mammal and habitat interactions. The modules are shown by different colours. The most centrally located node has an Eigenvector score closest to 1. **Figure S5.** Matrix of Goodman and Kruskal’s* τ* test for categorical variables describing small mammal and chigger species and the environment.

## Data Availability

All data has been made available in the article.
