# Supplementary material for: Orientia tsutsugamushi dynamics in vectors and hosts: ecology and risk factors for foci of scrub typhus transmission in northern Thailand
Source: Parasit Vectors. 2021 Oct 18;14:540. doi: 10.1186/s13071-021-05042-4 (PMC8524837; doi:10.1186/s13071-021-05042-4)
Supplement: Supplementary file 1 — Additional file 1: Table S1. Orientia tsutsugamushi PCR positivity by organ type and small mammal species. Table S2. Habitat classification scheme. Table S3. List of the most common plants identified at the study sites (1 = Ban Thoet Thai, 2 = Ban Song Kwair, 3 = Ban Mae Mon). Figure S1. Spatial distribution over time of all trap positions and O. tsutsugamushi-positive chigger pools, individual chiggers and small mammals for Ban Thoet Thai. This is the major town of the sub-district of Mae Fahluang district. Population: approx. 5,000; elevation: 550 m a.s.l.; mixed hill tribe ethnicities. Study site is located along a small river, encompassing rice fields, vegetable/fruit gardens, patches of fallow areas, degraded secondary forest and a few homes. Figure S2. Spatial distribution over time of all trap positions and O. tsutsugamushi-positive chigger pools, individual chiggers and small mammals for Ban Song Kwair. Home of human case is shown with star. This is an isolated small village in Mae Yao sub-district. Population: approx. 150; elevation: 650 m a.s.l.; Akha hilltribe village, predominantly with traditional wooden stilt homes. Study site is located beside a fast-flowing stream, at the head of the valley; it is surrounded by mixed secondary forest, teak plantation, fallow areas and bamboo groves, and there are patches of dry rice, corn and pineapple plantations. Figure S3. Spatial distribution over time of all trap positions and O. tsutsugamushi-positive chigger pools, individual chiggers and small mammals for Ban Mae Mon. Home of human case shown with star. The village is located in Huay Chomphu sub-district. Population: approx. 1500; elevation: 1200 m a.s.l.; mixed village of Akha and Lisu hill tribe people, predominantly concrete constructed homes. The village located on the steep slope of a ridge, surrounded by coffee, fruit and vegetable plantations, fallow areas and a small secondary forest. Figure S4. Non-quantitative nestedness matrix, bipartitie network, [file 13071_2021_5042_MOESM1_ESM.docx]

**Supplementary information**

Additional file: Table S1. *O. tsutsugamushi* PCR positivity by organ type and small mammal species

| **Small mammal species** | **Spleen** | **Liver** | **Lung** |
| --- | --- | --- | --- |
| **Rattus exulans** | **3** | **1** | **3** |
| **Rattus tanezumi** | **8** | **6** | **9** |
| **Rattus nitidus** | **1** | **1** |  |
| **Rattus andamanensis** | **5** | **1** | **4** |
| **Berylmys bowersi** | **1** |  | **1** |
| **Bandicota indica** | **3** |  | **4** |
| **Mus cookii** |  |  | **2** |
| **Total** | **21** | **9** | **23** |

Additional File: Table S2. Habitat classification scheme

| **Habitat resolution** | **Types** |
| --- | --- |
| **Low** | Lowland, upland, forest, settlement |
| **Medium** | Rice paddy field, rice paddy field/riverbank, riverbank, vegetable garden, vegetable garden/riverbank, village, village/riverbank, fallow, mixed secondary forest, teak plantation, fruit tree plantation, corn field, coffee plantation |
| **High** | See basic botanical survey, Supplementary material Table 2 |

Additional File: Table S3. List of the most common plants identified at the study sites (1 = Ban Thoet Thai, 2= Ban Song Kwair, 3= Ban Mae Mon)

| **Field Site** | **Plant species** | **Plant family** |
| --- | --- | --- |
| 1,2,3 | *Ageratum conyzoides* L. | **COMPOSITAE** |
| 1,2,3 | *Bidens pilosa* L. | **COMPOSITAE** |
| 1,2,3 | *Microstegium ciliatum* (Trin.) A.Camus | **GRAMINAE** |
| 1,2,3 | *Microstegium ciliatum* (Trin.) A.Camus | **GRAMINAE** |
| 1,2,3 | *Bambusa tulda* Roxb. | **GRAMINAE** |
| 1,2,3 | *Thysanolaena latifolia* (Roxb.) Honda | **GRAMINAE** |
| 3 | *Scleria terrestris* ( L. ) Fass. | **CYPERACEAE** |
| 3 | *Anomianthus dulcis* ( Dun.) Sincl. | **ANNONACEAE** |
| 3 | Genus unknown | **ANNONACEAE** |
| 3 | *Dalbergia foliacea* Wall. | **LEGUMINOSAE, PAPILIONOIDAE** |
| 1,2,3 | *Dendrocalamus barbatus* Hsueh & D.Z. Li | **GRAMINAE** |
| 2,3 | *Alpinia malaccensis* (Burm.f.) Roscoe | **ZINGIBERACEAE** |
| 1,2,3 | *Pennisetum polystachion* ( L. ) Schult. | **GRAMINAE** |
| 1,2,3 | *Saccharum spontaneum* L. | **GRAMINAE** |
| 1,3 | *Thyrsostachys siamensis* | **GRAMINAE** |
| 1,2,3 | *Imperata cylindrica* (L.) Raeusch. | **GRAMINAE** |
| 1 | *Pennisetum purpureum* Schumach. | **GRAMINAE** |
| 1,3 | *Pennisetum setaceum* | **GRAMINAE** |
| 1 | *Rottboellia exaltata* | **GRAMINAE** |
| 1 | *Senna siamea* | **LEGUMINOSAE, PAPILIONOIDAE** |

Additional File: Figure S1. Spatial distribution over time of all trap positions and *O. tsutsugamushi* positive chigger pools, individual chiggers and small mammals for Ban Thoet Thai. This is the major town of the sub-district of Mae Fahluang district. Population ~5,000. Elevation 550 masl. Mixed hill tribe ethnicities. Study site located along small river, encompassing rice fields, vegetable/fruit gardens, patches of fallow areas, degraded secondary forest and a few homes.


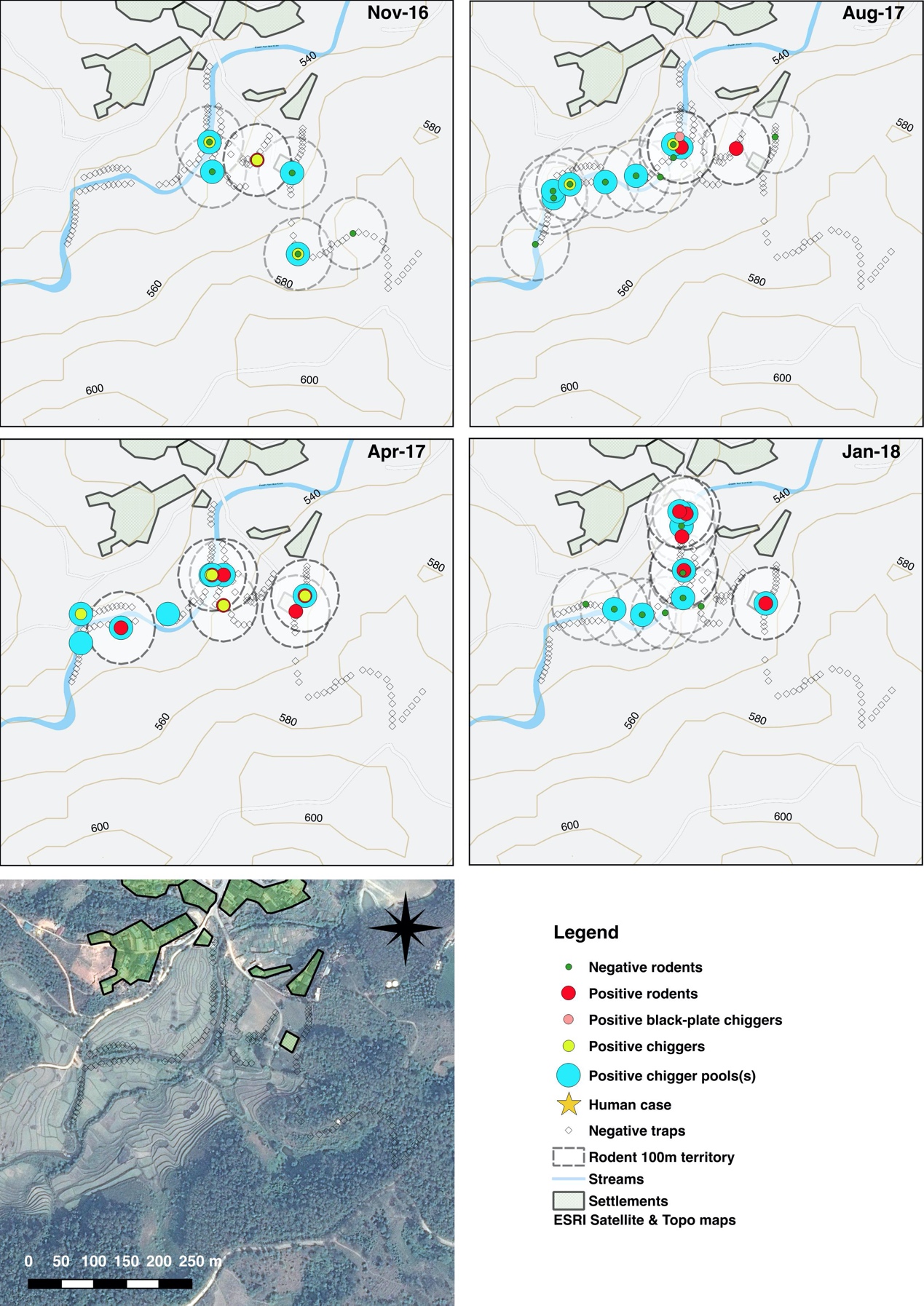


Additional File: Figure S2. Spatial distribution over time of all trap positions and *O. tsutsugamushi* positive chigger pools, individual chiggers and small mammals for Ban Song Kwair. Home of human case shown with star. An isolated small village in Mae Yao sub-district. Population ~150. Elevation 650 masl. Akha hilltribe village, predominantly with traditional wooden stilt homes. Beside fast-flowing stream, at the head of the valley. Surrounded by mixed secondary forest, teak plantation, fallow areas and bamboo groves. Patches of dry rice, corn and pineapple plantations.


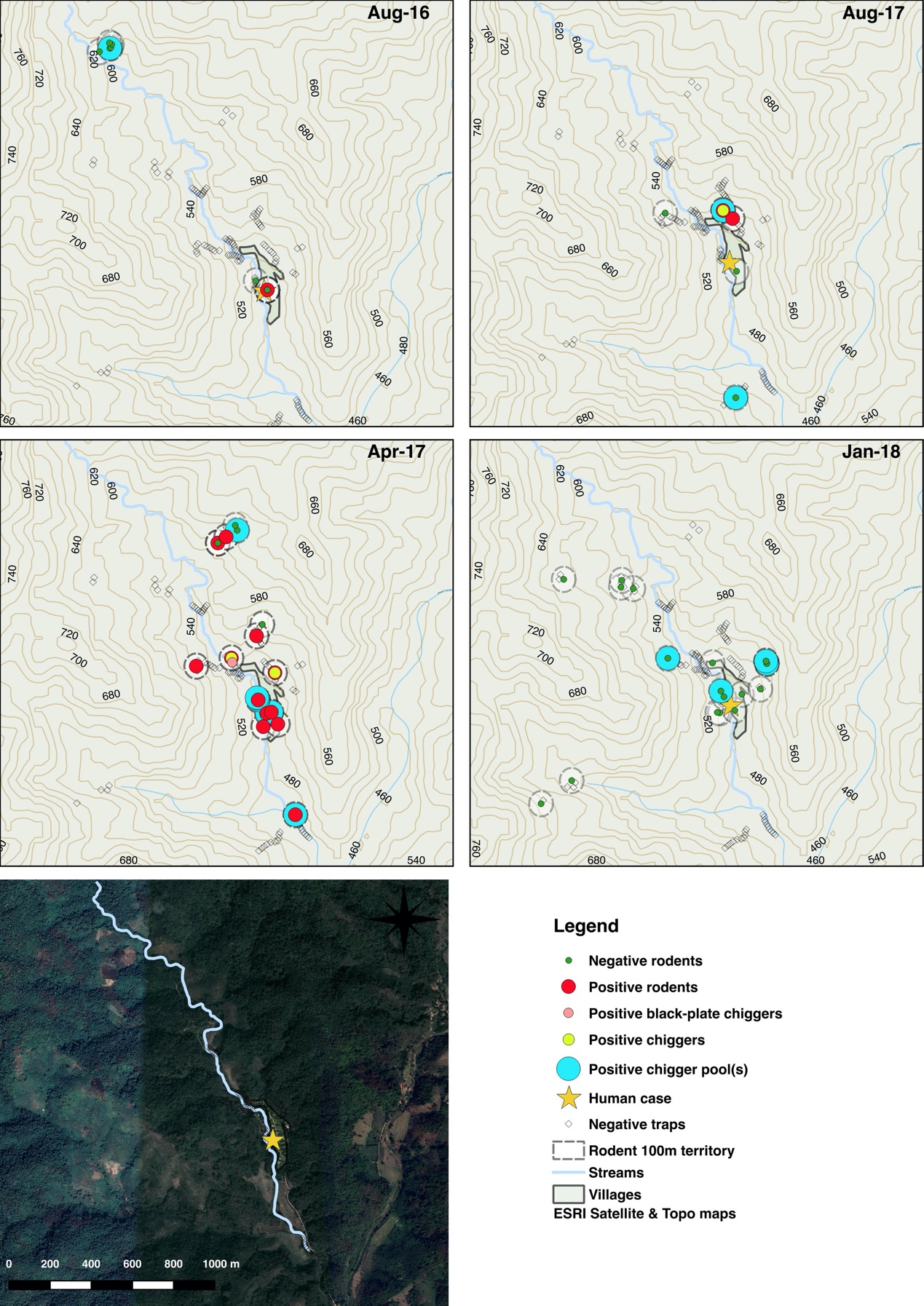


Additional File: Figure S3. Spatial distribution over time of all trap positions and *O. tsutsugamushi* positive chigger pools, individual chiggers and small mammals for Ban Mae Mon. Home of human case shown with star. The village is located in Huay Chomphu subdistrict. Population ~1,500. Elevation 1,200 masl. Mixed village of Akha and Lisu hill tribe people. Predominantly concrete constructed homes. Village located on the steep slope of a ridge, surrounded by coffee, fruit and vegetable plantations, fallow areas and a small secondary forest.


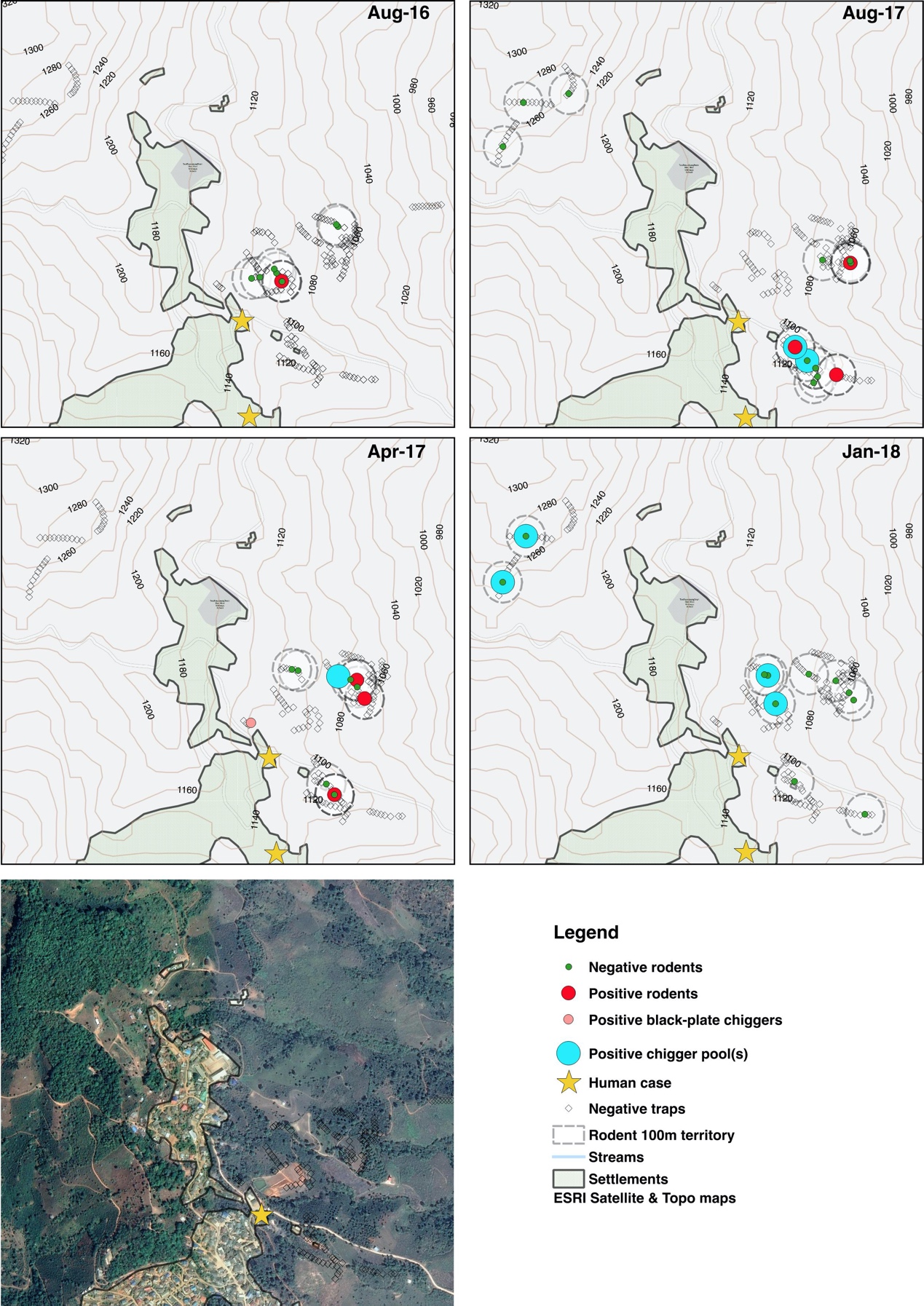


Additional File: Figure S4. Non-quantitative nestedness matrix (a), sub-community modules (b), bipartitie network (c) and unipartite network models for *O. tsutsugamushi* positive habitat (d) and chigger/small mammal (e) interactions. In the unipartite network, the modules are shown by different colours. The most centrally located node has an Eigenvector score closest to 1.

Additional File: Figure S5. Matrix of Goodman and Kruskal’s τ test, for categorical variables describing small mammal and chigger species and the environment.
